# Supplementary material for: Resilience in Lower Grade Glioma Patients
Source: Cancers (Basel). 2022 Nov 2;14(21):5410. doi: 10.3390/cancers14215410 (PMC9656661; doi:10.3390/cancers14215410)
Supplement: Supplementary file 1 [file cancers-14-05410-s001.zip › cancers-1949661-supplementary.pdf]

cancers

Table S1. Pearson-correlations of continuous focal variables with distress.

	Distress		
	Pearson Correlation	Sig. (2-tailed)	N
RS 13	-.491**	<.001	74
MOCA	-.075	.526	74
ECOG	.335**	.004	74
Internalized stigma	.271*	.019	74
Pain VAS 0-10	.460**	<.001	74
age	.000	.999	74
Time since diagnosis	-.082	.488	74
Social support	-.281*	.015	74

**. Correlation is significant at the 0.01 level (2-tailed). *. Correlation is significant at the 0.05 level (2-tailed).

Table S2. Spearman`s ϱ correlations of categorial focal variables with distress.

	Spearman's rho Distress		
	Correlation Coefficient	Sig. (2-tailed)	N
Gender	.138	.242	74
Education	-.119	.314	74
Relationship status	.289*	.012	74
Occupation	.018	.884	72
Chemotherapy	-.012	.918	74
radiation	-.125	.290	74

*. Correlation is significant at the 0.05 level (2-tailed.

Table S3. Distress as criterion and the focal variables as predictors.

Model		Unstandardized Coefficients		Standardized Coefficients	t	Sig.	95.0% Confidence Interval for B	
		B	Std. Error	Beta			Lower Bound	Upper Bound
1	(Constant)	2.312	3.650		.633	.529	-4.995	9.619
	Moca	-.010	.121	-.010	-.084	.933	-.252	.231
	ECOG	.721	.387	.323	1.863	.068	-.054	1.495
	Internalized stigma	.263	.861	.042	.305	.761	-1.460	1.986
	Pain	.357	.149	.340	2.392	.020	.058	.656
	Gender	.563	.605	.109	.931	.356	-.648	1.773
	education	-.698	.573	-.136	-1.216	.229	-1.846	.450
	age	-.020	.032	-.084	-.620	.538	-.083	.044
	ocupation	1.776	.836	.335	2.125	.038	.103	3.450
	Time since diagnosis	-.064	.116	-.063	-.552	.583	-.296	.168
	chemotherapy	.465	1.017	.091	.458	.649	-1.570	2.500
	Radiation therapy	-.585	1.000	-.112	-.585	.561	-2.586	1.416
	Social support	-.065	.297	-.025	-.219	.828	-.659	.529
	Relationship status	1.913	.692	.305	2.765	.008	.528	3.298

a. Dependent Variable: Distress.
